# An accurate strategy for pointing the key biocatalytic sites of bre2691A protein for modification of the brevilaterin from *Brevibacillus laterosporus*

**DOI:** 10.1186/s12934-022-01918-x

**Published:** 2022-09-19

**Authors:** Panpan Han, Zhou Chen, Yangliu Liu, Aijin Ma, Siting Li, Yingmin Jia

**Affiliations:** School of Food and Health, Beijing Technology and Business University, No.33 Fucheng Road, Haidian District, Beijing, 100048 China

**Keywords:** Biocatalytic, Adenylation domain, Cationic antimicrobial peptide, *Brevibacillus laterosporus*

## Abstract

**Background:**

Brevilaterin A-E, a novel class of multi-component cationic antimicrobial lipopeptides, were biosynthesized by a non-ribosomal peptides synthetase (NRPS) in *Brevibacillus laterosporus*. However, the antimicrobial abilities of different brevilaterin components varied greatly, and this multi-component form was impeding the scale production of the excellent component, and a little information about the brevilaterin biosynthesis mechanism was available to apply in brevilaterin design modification. In this study, we used an accurate strategy that revealed the reason for producing multi-component was the substrate selectivity of bre2691A protein being not enough specific and pinpointed the key design sites to make the specificity of bre2691A enhanced.

**Results:**

Bioinformatic analysis revealed that the biocatalytic site of bre2691A, which was an adenylation domain catalyzed and recognized methionine, leucine, valine and isoleucine and thus introduced them into brevilaterins and caused different components (brevilaterin A-E), was consisted of A1 ~ A10 residues named specificity-conferring code. Coupling molecular docking simulations with mutation studies identified A2 and A7 as critical residues, where determined substrate-specificity and impacted activity. The in virto activity assay showed that the A2 mutant (G193A) would lose activity against methionine and have no effect on the other three amino acids, the A7 mutant (G285C) would enhance the catalytic activity against four substrates, especially against leucine at almost a double activity. When the A2 and A7 residues were synchronously mutated, this mutant would be more focused on recognizing leucine.

**Conclusions:**

An accurate strategy that combined with bioinformatics and site-directed mutation techniques revealed the pivotal site A2 and A7 positions of bre2691A protein that could be used to design and modify brevilaterins, thus further providing a reasonable direction of genetic engineering for *Brevibacillus laterosporus*. A deeper understanding of the function of crucial residues in the adenylation domain would make it get more accurate and highly efficient design and more fully utilized. Furthermore, it would contribute to biotechnological applications, namely for the large centralized synthesis of antimicrobial peptides, or for the optimization of their production.

**Graphic Abstract:**

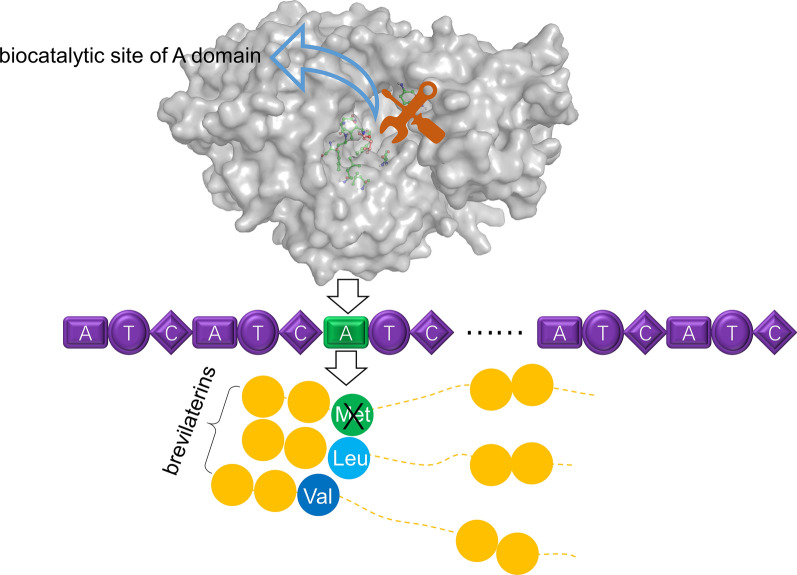

## Background

The microorganism would produce a variety of secondary metabolites, of which the bioactive substances drew great attention from researchers and had important potential application value. Many bioactive substances, such as natamycin [1], enduracidin, and rapamycin [2], were developed and applied in food, animal husbandry, and medicine field. Most of them were synthesized by the non-ribosomal peptide synthetases (NRPS) or NRPS hybridized with polyketide synthases (NRPS/PKS). The NRPS system was composed of an array of distinct modules, and the module was further divided into various functional domains, which mainly were adenylation (A) domain, peptidyl-carrier protein (PCP) domain, condensation (C) domain and other tailor domains [3]. In NRPS biosynthesis, every domain had its responsibility. the A domain was the start of the module and activates the carboxy group of substrates to be an aminoacyl adenylate, which was the activated state of substrate [4]. The *holo* form of PCP domain [5] could guide the aminoacyl adenylate state of substrate to the C domain by a covalent bond. Finally, an amide bond was built at the active site of the C domain to catalyze the peptidyl chain elongation reaction [6].

From the above, the substrate specificity and enzyme activity of the A domain made a large contribution to determining the chemical structure and production of non-ribosomal peptides. Understanding the information of substrate recognized mechanism of A domain was certainly important for rational genetic engineering of NRPS assembly lines to generate novel peptides and increase production. In the biochemical reaction process of the A domain, the nucleophilic substitution reaction with ATP happened in the active site of A domain, when the substrate was recognized. The creation of aminoacyl adenylate state of substrate needed the carboxy group of substrate and ATP to be precisely fixed in the appropriate Bürgi–Dunitz angle for the next step reaction that nucleophilic attack of the carboxylic acid to an α-phosphorous atom of ATP [4]. This process would be acted at the substrate-binding pocket constituted by specificity-conferring code (A1-A10 position), which consisted of ten key residue sites and determined substrate selectivity of the A domain [7]. Subsequently, the researchers focused on this code of A domains from different microorganisms. Fumitaka Kudo [4] summarized them and found that the codes derived from different microorganisms were discrepant, even though they recognized the same substrate. Hence, decoding this code was of great concern to predict the substrate-selectivity of A domain. Hitherto, the codes from *Streptomyces spp *[8–11], *Pseudomonas spp *[12, 13] and a few *Bacillus spp* [14]had been studied. Theoretically, the substrate-specificity of A domain could be changed by site-directed mutagenesis, and then it was successful in regulating the production of non-ribosomal peptides and providing a design direction for the artificial modification of amino acid composition in the structure of non-ribosomal peptides.

Recently, due to the proliferation of antibiotic-resistant bacteria, in those non-ribosomal peptides, the antimicrobial peptides may be able to serve as alternative therapeutic to antibiotics and drew great public attention. Among them, the cationic antimicrobial peptides were well-known for their highly-efficient and broad-spectrum antimicrobial ability and particular membranolytic mechanism [15]. However, they were usually produced in the form of multiple components, which were mixtures biosynthesized by the same NRPS system and had a similar amino acid sequence but several amino acid differences, namely they were all derivates of the same parent small-molecule peptide, for instance, gramicidin A-C [16], tyrocidine A–C [17] and et.al. Moreover, *Brevibacillus laterosporus* as a bioresource for producing a novel class of cationic antimicrobial peptides had attracted much attention. Many excellent cationic antimicrobial peptides produced by them were reported, notably loloatin A–D [18], bogorol A-E [19], brevilaterin A-E [20], laterocidin 1–10 [21], brevicidine 1–2 [22] and so on. Though only several amino acids were different in the structure of different components, their antimicrobial activities varied greatly. The high-efficiency component was the most cost-effective product, but this form led to the strain can not concentrate on producing the best one, and the other components would cause trouble for the later extraction and purification work. This was the sticking point to restrict the commercialization of antimicrobial peptides derived from microorganisms. Therefore, exploring the reason for producing multi-component and generating a strategy to rationally reduce the number of components were important for the commercial production of antimicrobial peptides.

In this study, an A domain named bre2691A was analyzed to be the crux of forming multiple brevilaterin components. A strategy that combed the bioinformatics analysis with site-directed mutagenesis revealed the function position for highly efficient transformation substrate selectivity of bre2691A, and then provided theoretical support for future artificial design and regulating the production of antimicrobial peptides.

## Materials and methods

### Bioinformatic analysis of bre2691A

The multiple sequence alignment among bre2691A[23] and other fourteen A domains was performed by CLUSTALW 2.1 (https://www.genome.jp/tools-bin/clustalw, system default parameter settings) and the conserved core motifs among them were found by Multiple Em for Motif Elicitation 5.4.1 (https://meme-suite.org/meme/tools/meme, select the motif discovery mode: Classic mode, select the number of motifs: 10) [24]. The construction of phylogenetic tree about them was built by MEGA-X (version 10.2.6) [25] using the Neighbor-Joining algorithm (No. of Bootstrap Replications: 1000). The homology model of bre2691A was constructed by SWISS-MODEL [26] (https://swissmodel.expasy.org/), taking the cocrystal structure of SrfA-C with ligands (leucine, SO_4_) (PDB ID:2vsq.1) as a template. Molecular docking of bre2691A to leucine was performed through AutoDock vina 1.1.2 (default parameter settings). The figures were prepared using PyMOL 2.4.1 [27].

### Preparation of recombinant mutants

For the construction of four mutants G193A, L235F, G285C and a tri-mutant, which simultaneously made G193A, L235F and G285C in one mutant, the pET21b-bre2691A plasmid was used for site-directed mutagenesis. Site-directed mutagenesis was performed with a Q5 site-directed mutagenesis kit (NEB, (Beijing) LTD) using the following oligonucleotides: G193A, F: 5′-ctaattatgcttttgatgcttctacctttgatatatacag-3′, R: 5′-gtatatatcaaaggtagaagcatcaaaagcataattagac-3′; L235F, F: 5′-tatcacggtagctttcctgacaacctctctattcaatacg-3′, R: 5′-atagagaggttgtcaggaaagctaccgtgatattgctatc-3′; G285C, F: 5′-gggcgcctagtaaattgctatggtccgacagaaacaacgg-3′, R: 5′-gtttctgtcggaccatagcaatttactaggcgcccctcac-3′. The mutants were sequenced to verify their correctness by Taihe Biotechnology Co., Ltd.

### Expression and purification of mutants

All mutants were overexpressed in *E.coli* BL21(DE3) *ΔybdZ* grown in an LB culture medium supplemented with ampicillin at 50 μg/mL. Cultures were grown at 37 °C with 200 rpm to OD_600_ = 0.6 ~ 0.8, and IPTG of 0.1 mM was added and induced at 16℃ for 18 h. The cells were harvested by centrifugation (8000 rpm, 10 min, 4 °C), resuspended in lysis buffer (50 mM Tris–HCl, pH 8.0, 200 mM NaCl, 20 mM imidazole, 0.2 mM TCEP, 10% glycerol), and lysed by sonication on ice.

His-tagged proteins were purified by using Ni–NTA agarose (ÄKTA pure protein purification system, Cytiva) following the supplied protocols. The cleared cell lysate following sonication and centrifugation was directly applied onto a column packed with Ni-NTA agarose. After washing with washing buffer (50 mM Tris-HCl, 200 mM NaCl, 0.2 mM TCEP, pH 8.0, 10% glycerol) containing 20 mM imidazole, the protein was eluted with the elution buffer (50 mM Tris-HCl, 200 mM NaCl, 200 mM imidazole, 0.2 mM TCEP, pH8.0, 10% glycerol). Purified proteins were concentrated and buffer exchanged into a storage buffer (50 mM Tris-HCl, 200 mM NaCl, 0.2 mM TCEP, pH = 8.0, 10% glycerol). Protein concentrations were determined by the BCA protein assay kit (BN27109, Biorigin, Beijing) using BSA as a standard.

### In vitro characterization of the mutants

The colorimetric pyrophosphate production assays were used to monitor adenylation of the mutants of bre2691A. They were performed in triplicate 100 μL reactions containing 50 mM Tri-HCl (pH7.5), 200 mM NaCl, 10 mM MgCl_2_, 0.2 mM TCEP, 10% glycerol, 1 mM ATP, 0.2 mM 2-amino-6-mercapto-7-methylpurine ribonucleoside, 1U/mL purine nucleoside phosphorylase, 0.03U/mL inorganic pyrophosphatase, 1 mM substrates. Reactions were set in 96 microwell plates and were started by addition of 1 μM the mutants of bre2691A proteins.

## Results and discussion

### Analysis of the reason for brevilaterin biosynthesis system to produce multi-component peptides

In the non-ribosomal peptides biosynthesis, the A domain was of note in determining the final chemical structure. Not only amino acids, but also unnatural amino acids, hydroxyl acids and keto acids would be utilized, which would make structure more complex and biological activity more diverse as α-hydroxyl-isoleucine (α-HIL), and ornithine in brevilaterin [20]. In general, the A domain had a strong substrate-specificity. Most A domains could identify only one substrate, whereas there was the substrate selectivity of some A domains that were not very specific. Therefore, when the NRPS synthesized their products, some of A domains would simultaneously introduce different amino acids into the basic structure and produce a variety of structural analogues. Hence, identifying which A domain in their NRPS biosynthesis system could simultaneously recognize multiple amino acids which were the structural differences of the multicomponent antimicrobial peptides would help resolve such problems at source. After that, realizing the substrate-specific recognition mechanism of that A domain was significance to modify and design the peptide structure, and resolve the problem of strains producing multi-components.

A previous study on all A domains in the brevilaterin biosynthesis had shown that the bre2691A was an A domain that simultaneously recognized four amino acids, which were methionine, leucine, valine and isoleucine. The four amino acids were structural differences of brevilaterin A-E [20]. Consequently, combining the amino acid sequence of brevilaterins, bre2691A was analyzed to be in charge of introducing the third residue of brevilaterin A-E and the crux for biosynthesizing multiple components. Thus, the analysis of substrate-specificity recognition mechanism of bre2691A and finding the key biocatalytic sites were essential for artificial designing and regulating the production of antimicrobial peptides.

### Identification of the key biocatalytic site in bre2691A to activate substrate

Many studies had displayed that a specificity-conferring code contained in A domain was related to the substrate-specificity recognition. This code built a substrate-binding pocket namely biocatalytic sites to accommodate the substrates and further active them. However, the codes of most A domains that came from *Streptomyces* spp and few *Bacillus* spp were reviewed and analyzed. Due to minority cationic antimicrobial peptide synthease systems from *Brevibacillus laterosporus* being reported, the research on the specificity-conferring code from the above system was seldom.

**Fig. 1:**
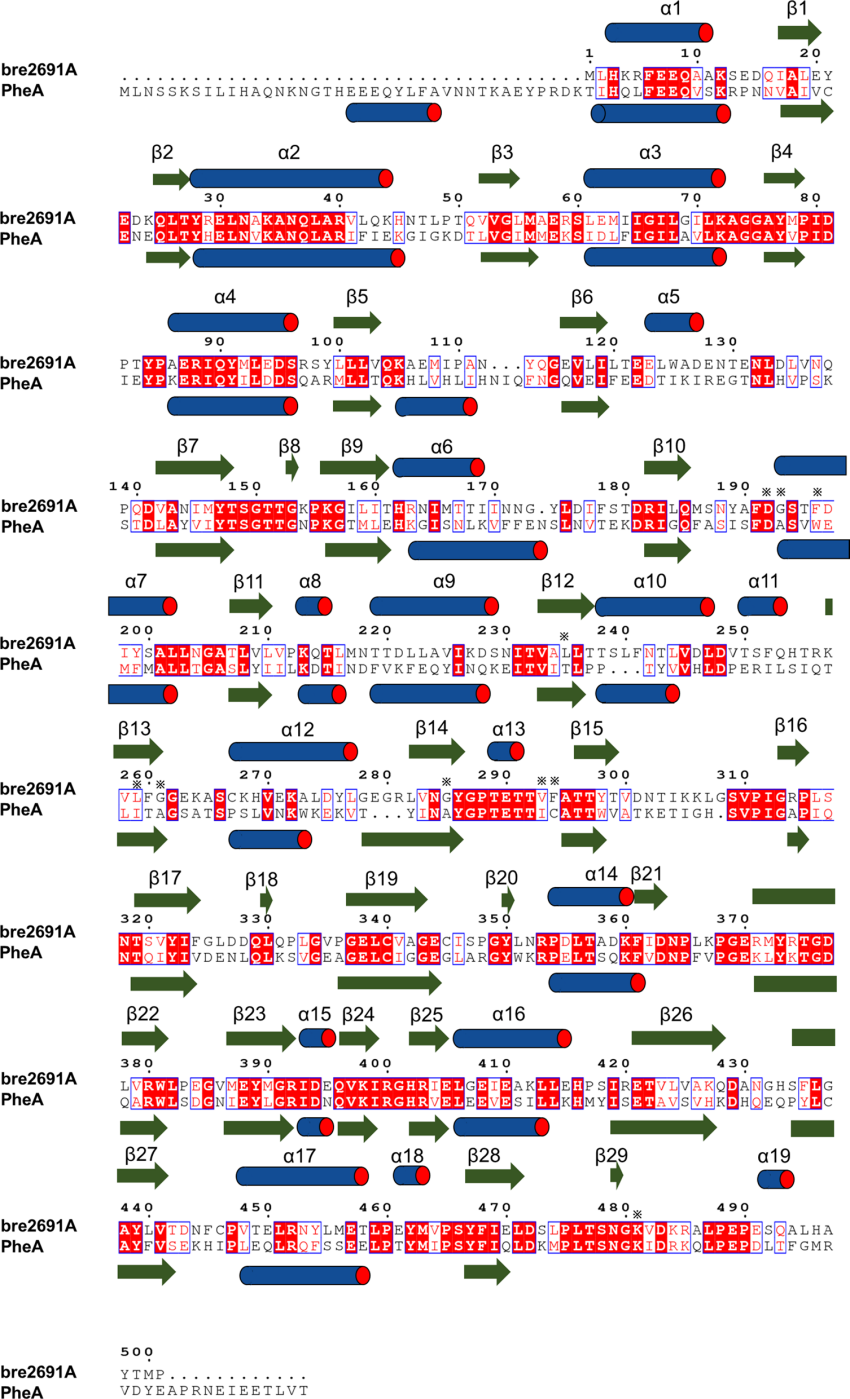
Amino acid sequence comparison of bre2691A with PheA. Specificity-conferring code residues were marked with an asterisk. The secondary structural elements of bre2691A and PheA were indicated by bars above or below the sequence. The conserved positions were shown in red

**Fig. 2:**
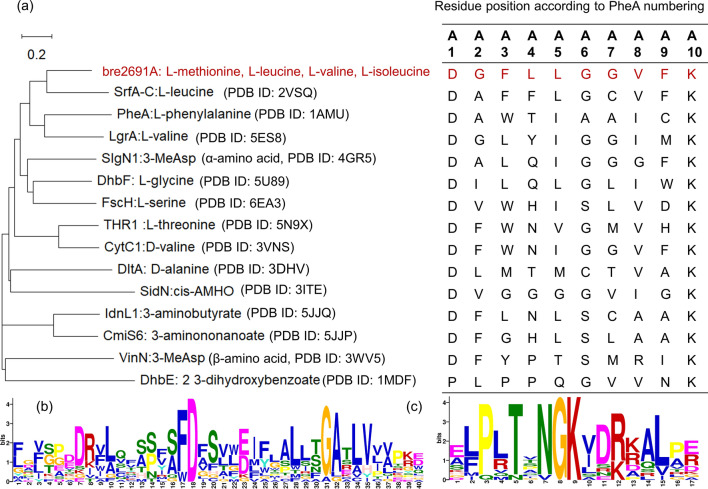
Phylogenetic analysis of A domain. **a** The sequences of the following 15 A domains were used for phylogenetic analysis and their specificity-conferring codes. *B. laterosporus* Bre2691A (this study), *B.subtilis* SrfA-C [29], *B. brevis* PheA [14], *B. parabrevis* LgrA [30], *Streptomyces lydicus* SlgN1 [11], *Geobacillus sp*. Y4.1MC1 DhbF [31], *Thermobifida fusca* YX FscH, *Streptomyces* SP.OH-5093 THR1, *Streptomyces sp*. CytC1, *B. cereus* ATCC 14,579 DltA [35], *Epichloe festucae var. lolii* SidN [36], *Streptomyces sp*. ML694-90F3 IdnL1, *Streptomyces sp*. MJ635-86F5 CmiS6, *Streptomyces halstedii* VinN, *B. subtilis* DhbE [37]. **b** the specificity-conferring codes from 15 A domains according to the PheA numbering. **c** and **d** Conserved core motifs in fifteen A domains. **b** the location of A1, A2 and A3 positions showed at number 18, 19 and 22. **c** the location of A10 position showed at number 9

Though amino acid sequence comparison of bre2691A with PheA (Fig. 1), which recognized L-phenylalanine as substrate and was the A domain that first proposed the specificity-conferring code theory based on its crystal structure. The specificity-conferring code of bre2691A was pointed out and consisted of ten residues, which were Asp^192^ (A1), Gly^193^ (A2), Phe^196^ (A3), Leu^235^ (A4), Leu^259^ (A5), Gly^261^ (A6), Gly^285^ (A7), Val^293^ (A8), Phe^294^ (A9), Lys^481^ (A10). In bre2691A, only A1 and A10 were same as PheA. In addition, the comparison of the structure with the phylogenetic analysis of bre2691A and the other A domains (Fig. 2a). Researches showed that the α-amino and the α-carboxylate groups of substrate amino acid were respectively stabilized by electrostatic interactions with A1 and A10 position, which were Asp^192^ and Lys^481^ in bre2691A as observed in other A domains and played a general role in catalysis (Fig. 2b). These residues were located in the conserved core motifs (Fig. 2c, d), with the latter being found in the carboxy-terminal subdomain. the other residues of the code in bre2691A were totally unlike PheA and maybe participate in recognition to side chain groups of substrate. The other residues created a specificity pocket and were found surrounding the active site where the substrate and ATP bind and hydrolysis [14]. Whether changing the type of residues here would alter the substrate specificity of the A domain, and towards which kind of substrate would get more attention. It was needed to be explored.

### Functional analysis of biocatalytic site residues in bre2691A

**Fig. 3:**
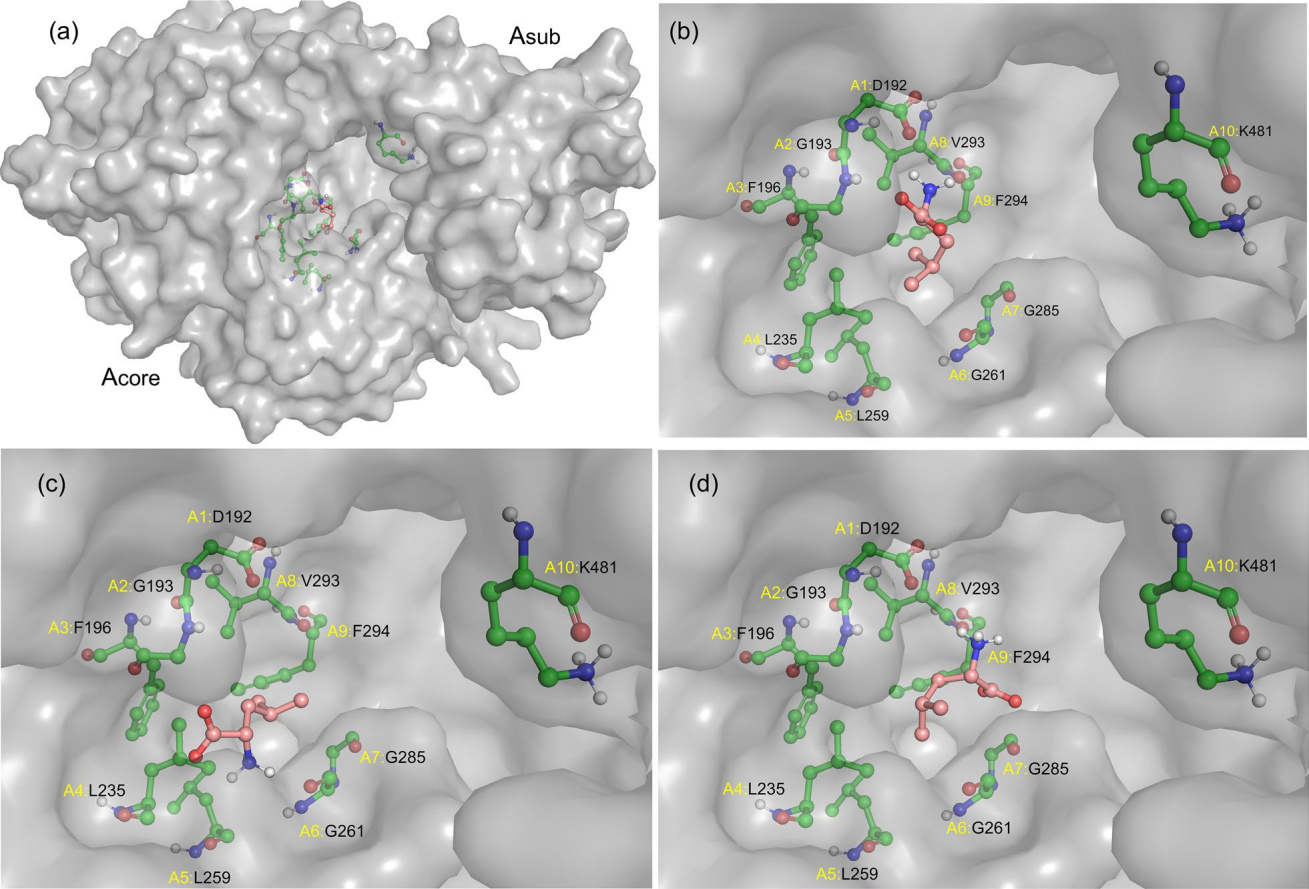
Substrate specificity recognition process of bre2691A. **a** Three dimensional structural model of bre2691A, the specificity pocket of A domain contacting leucine in Acore, Asub was in “closed” state, after A domain captured leucine. **b**, **c** and **d** The leucine (salmon stick) entered the specificity pocket in different angels. The specificity-conferring code were shown as green sticks

The researches on the antimicrobial mechanism of peptides suggested that the leucine and lysine rich antimicrobial peptides indicated an enhanced antimicrobial activity without hemolysis [28]. This was due to the increase hydrophobic region by leucine substitution and the net positive charge by lysine substitution, which prompted the antimicrobial peptide to bind with the cell membrane, further made the cellular content leak out and eventually killed the germs. Therefore, the preference of A domain to select leucine would be more contributed to artificial design the novel antimicrobial peptides and those analogues. In this study, the bre2691A was more like to activate the methionine. Whether it could be made more sensitive to leucine? Though uniting the comparison of the structure with the phylogenetic analysis of bre2691A and the others (Fig. 2a), which came from different microorganism and had an activity against diversified substrates, it was found that bre2691A respectively had 48.18%, 43.96% and 39.59% homology to SrfA-C [29], LgrA [30] and DhbF [31] and was more approached to SrfA-C. Subsequently, the three dimensional structure of bre2691A was built based on the SrfA-C (Fig. 3a). The result demonstrated that bre2691A was composed of two parts (Acore and Asub). Indeed, the active site was located at the interface between the folding domain (Acore) and roration close (Asub) described as a cleft between them (Fig. 3a). When the nucleophilic substitution for adenylate was reacted between substrate and ATP, the Asub part would acted like a door. The specific performance is the Asub would rotated to the state as shown in the Fig. 3a, in order to the binding of the substrates and the postadenylation state that locked and protected the product. In bre2691A, the specificity pocket contained in Acore exhibited a wide space allowed leucine to enter in different angles (Fig. 3b, c, d). This wide space would tolerate many kinds of substrates, maybe it was the reason that bre2691A manifested four amino acids. Whether it could be more targeted to activate leucine by shallowing this space, and which one position was function?

From phylogenetic analysis of bre2691A and other A domains (Fig. 2a, b), it found that bre2691A had a similar specificity conferring code to SrfA-C, and they both had activity against leucine. There were only three positions presented different amino acids. Hence, those different positions A2, A4 and A7 possibly were related to recognize leucine, which respectively were glycine, leucine and glycine in bre2691A and alanine, phenylalanine and cysteine in SrfA-C. The similar situation also appeared between IdnL and CmiS6 [8]. IdnL1 had a bulky Leu^220^ (A3) located close to the terminal methyl group of 3-aminobutanoate of the trapped acyl-adenylate intermediate to construct a shallow specificity pocket. In contrast, CmiS6 possessed Gly^220^ (A3) at the corresponding position to accommodate 3-aminononanoic acid. Therefore, by shallowing the specificity pocket would modify substrate specificity [8]. Likewise, the THR1 and CytC1 [10], there were also three different residues but at the position A5, A7 and A9. Parsing the crystal structure of THR1, A5 position did not establish direct contacts with the substrate but contributed to fix the position of A2 and A9 that were crucial for threonine recognition within the tightly packed protein core and could also be isoleucine or leucine. The A7 was a crucial position, due to it both interacted with the threonine and adenine. From the structure of SrfA-C, the A2 and A4 were recruited in the way of hydrogen bond with the α-amino group of leucine and hydrophobic interaction with the side-chain of leucine. Whereas the A4 position called ‘wobble’-like positions did not interact directly with the substrate and revealed an elevated variability throughout all codons determined, even in different A domains that recognize the same substrate [7]. On the based of above information, which one position among A2, A4 and A7 were truly helpful for bre2691A to be tend to recognize leucine.

### Identification of the key function position of biocatalytic site in bre2691A

**Fig. 4:**
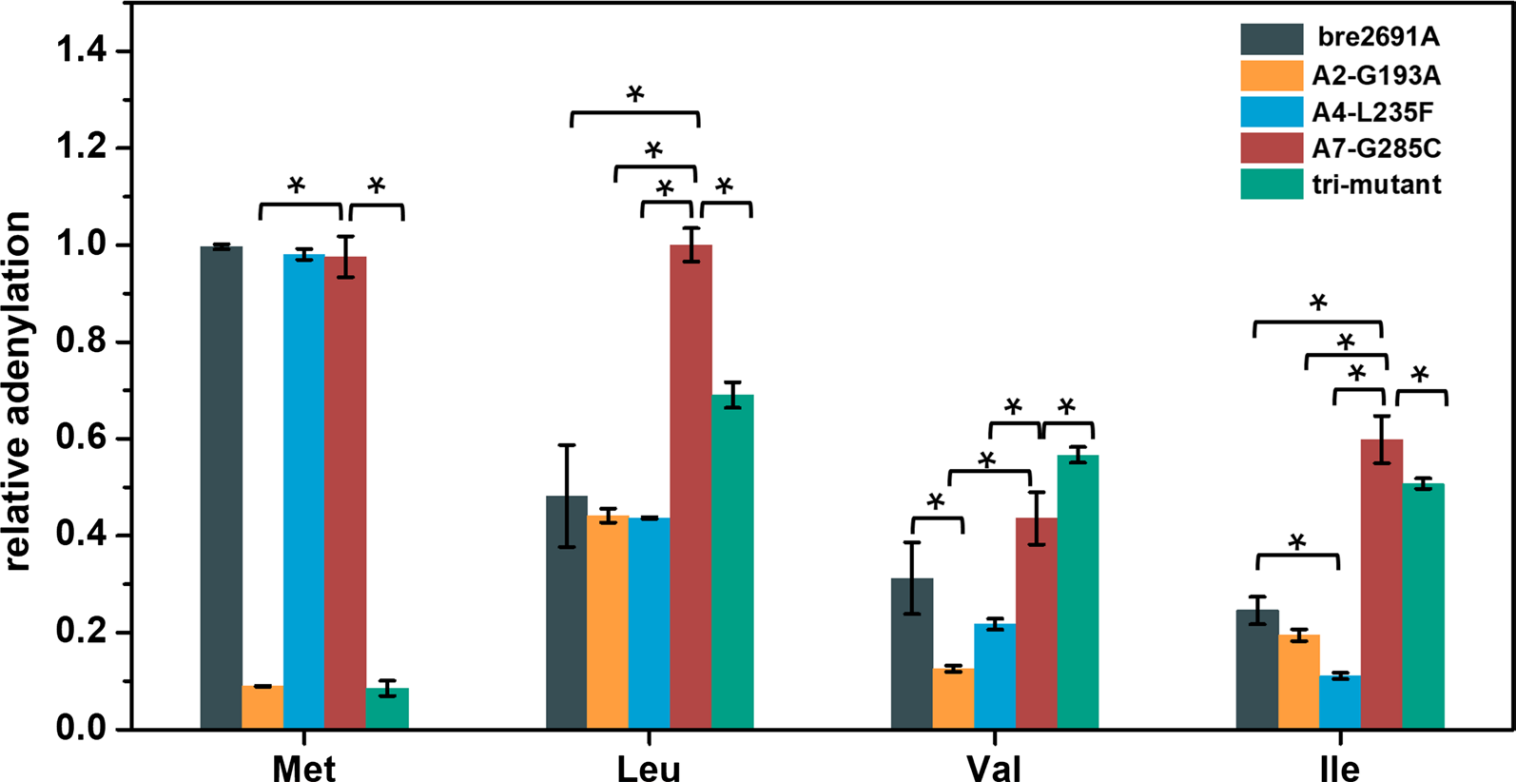
The relative adenylation activity of wild type and mutants of bre2691A. Each sample contained three replicates and tested in triplicate. The statistical analysis was preformed by PASW Statistics 18.0 (SPSS Inc., Armonk, NY, USA) using one-way analysis of variance (*P* < 0.05). The figure was prepared by Origin 8.1

Decisively, based on the above results, the mutational studies on bre2691A further revealed the truly function of A2, A4 and A7 position (Fig. 4). First, the Gly^193^ (A2) position was mutant to alanine, according to SrfA-C. The G193A mutant inconceivably had hardly any activity against methionine, but no effect on the other three amino acids. However, G193A has a dramatically decreased activity against valine, and a moderate reduction for isoleucine. It seemed to because of the different hydrophobicity between glycine and alanine. A similar phenomenon was also found in CmiS6, in which Gly220/Leu312 indirectly made the appropriate hydrophobic atmosphere to accommodate the appropriate length of side chain, the CmiS6 G220L mutant could recognize 3-ABA as substrate, but had lost the affinity for 3-ANA [8]. When the A domain acquired substrate, the right hydrophobic environment seemed likely to be needed. In the 3.3, we talked about that the A2 and A4 were recruited in the way of a hydrogen bond with the α-amino group of substrate hydrophobic interaction with the side-chain of substrate [29]. Therefore, when A2 was mutated from glycine to alanine, the hydrophobicity would be changed, G193A mutant would hardly acquire methionine, and also had different degrees of influence on activity levels against the other three amino acids. In addition, because valine had a smaller side chain and was harder to be captured than isoleucine, the G193A mutant had a deeper impact on the recognizing valine. The same phenomenon was also presented in the other two A domains belonged to same biosynthesis system from *Brevibacillus laterosporus*, which were bre2684A and bre2692A [23]. They both recognized lysine and ornithine, but the bre2684A preferred to adenylate lysine and the bre2692A got more attention to ornithine, and there was very low activity against the other of the two substrate. Comparing their specificity-conferring codes, only A2 position was respective amino acid. Corresponding, alanine was in bre2684A and serine was in bre2692A. From the above, the A2 was a crucial position that modified substrate specificity. Its principle was possibly uniformed to IdnL, namely shallowing the specificity pocket through a relatively bulky amino acid exactly as leucine in IdnL, alanine in G193A mutant, serine in bre2692A.

Subsequently, the other two of the three positions were also studied by mutation research. The A4 position (Leu^235^) was mutated to phenylalanine. There was no significant change compared L235F mutant with the wild bre2691A, except a gentle reduction for isoleucine. This position also be called ‘wobble’-like position and did not interact directly with the substrate and revealed an elevated variability [7]. Maybe this was the reason that L235F did not show an obvious change. Additionally, some cases had similar results, for instance, the GetJA_4_ and GetMA_5_ [32], which showed a similar activity against tryptophan, tyrosine, histidine, lysine, arginine and glycine but only had a difference at A4 position that serine and leucine respectively. As for A7 position in bre2691A, just like the previous analysis of the THR1 [10], the A7 residue (Gly^285^) was extremely crucial position in specificity-conferring code. The G285C mutant showed an enhanced activity against recognized four substrates, especially against leucine at almost double activity. However, this result was contrary to VinN, the M323G mutant of VinN completely lost activity [33]. This two results both indicated that A7 position had great influence on activating substrates. From the structure, VinN revealed that Met^323^ (A7) did not directly interact with the 3-MeAsp substrate but was likely to modulate the conformation of the Lys^330^ (A8) and Arg^331^ (A9) side chains by steric effects [33]. Similarly, the G285C mutant showed an analogical site here (Fig. 3). This site space would be made stronger through steric effects and easier to trap substrates, and a smaller side chain of glycine seemed like not enough to create a spatial effect based on the activity results of the mutants VinN M323G and bre2691 G285C.

From the above, these positions were important to enhance activity and modify substrate. When all three sites were mutated simultaneously, this tri-mutant manifested the preferring activity against leucine, which was an amino acid got more attention in the research on artificial peptide design. This tri-mutant almost lost activity against methionine, whereas it was employed in an enhanced activity against leucine. This demonstrates that A2 and A7 position, which were contained in specificity-conferring code from *Brevibacillus laterosporus*, were of great importance to recognize substrates. From the analysis of mutants G193A and G285C, the A2 position would likely provided a relatively suitable hydrophobic environment for leucine and not for methionine, and also shallowed this substrate-binding pocket. Additionally, the cysteine at A7 position modulate the conformation of the Val^293^ (A8) and Phe^294^ (A9) side chains by steric effects and made it easier to capture the substrate. Therefore, when they were simultaneously mutated these two site, this A domain exhibited a relatively enhanced activity against leucine and a lost activity against methionine. Moreover, the similar situation had been reported by David L. Niquille [34]. By changing the amino acid of the key position and constructing a mutant, the A domain TycB from *Brevibacillus parabrevis* was succeed to modify their substrate and select more substrates than it had before. This operation resulted that this NRPS molecule machine produced tyrocidine A and other 11 analogues. Then it was more easily to screen out more excellent antibacterial peptides. Whereas the brevilaterin biosynthesis system itself produce multiple components. For detailed characterization and subsequent chemical modification of the bioactive core, biosynthesis would be scaled up for producing preparative amounts of a pure single component. Therefore, narrowing the substrate range was more beneficial to practical production of a single brevilaterin component. And the tri-mutant would support for brevilaterin biosynthesis system more precisely concentrated to produce component E.

In brief, the A2 position did not contact with substrate, but would shallow the specificity pocket space and have an effect on specificity. The A7 position would probably interact with the substrate and adenine then affected the activity. When they were mutated, the mutant would completely change its substrate preference. Thus the substrate specificity of A domain could be altered through site-directed mutagenesis.

## Conclusion

Overall, we revealed that the reason for brevilaterin biosynthesis system producing multi-components was the substrate selectivity of bre2691A was not specific. The A domain had more than one natural specific substrate and exhibited partiality. The substrate selectivity of A domain was determined by the specificity-conferring code, and it could be designed by parsing the function of the key position in the code. When the responsible key catalytic residues were discovered and mutated into suitable amino acids, the A domain would change the substrate selectivity and be more precisely concentrated to recognize substrate that more benefited to bioactive. However, the locations of key biocatalytic residues in the specific codes of A domain from different microorganisms were inconsistent, and the functional effects were also different. This research generated an accurate strategy to quickly point that the A2 and A7 position in specificity-conferring code as the key function residues that could change the substrate selectivity of bre2691A, and contributed to a deeper understanding about the role of key positions on the substrate recognition mechanism of an A domain from *Brevibacillus laterosporus*. According to this research, the A domain could get a more accurate design and be more fully developed and utilized. Indeed, a deeper knowledge of the function of crucial positions related to the substrate recognition mechanism of the A domains, which were the gatekeepers of nonribosomal assembly lines, was interesting for biotechnological applications, namely for the large centralized synthesis of novel antimicrobial peptides by combinatorial biosynthesis, or for the optimization of their production.

## Data Availability

All data generated during this study are included in this article, and all material is available upon request.

## Abbreviations

NRPS: Nonribosomal peptide synthetase
PKS: Polyketide synthases
A domain: Adenylation domain
PCP-domain: Peptidyl carrier protein domain
C-domain: Condensation domain
α-HIL: α-Hydroxyl-isoleucine
